# Dynamic shrinkage in time‐varying parameter stochastic volatility in mean models

**DOI:** 10.1002/jae.2804

**Published:** 2021-01-06

**Authors:** Florian Huber, Michael Pfarrhofer

**Affiliations:** ^1^ Department of Economics Salzburg Centre of European Union Studies University of Salzburg Mönchsberg 2A Salzburg 5020 Austria

**Keywords:** inflation forecasting, inflation uncertainty, real‐time data, replication, state‐space models

## Abstract

Successful forecasting models strike a balance between parsimony and flexibility. This is often achieved by employing suitable shrinkage priors that penalize model complexity but also reward model fit. In this article, we modify the stochastic volatility in mean (SVM) model by introducing state‐of‐the‐art shrinkage techniques that allow for time variation in the degree of shrinkage. Using a real‐time inflation forecast exercise, we show that employing more flexible prior distributions on several key parameters sometimes improves forecast performance for the United States, the United Kingdom, and the euro area (EA). Comparing in‐sample results reveals that our proposed model yields qualitatively similar insights to the original version of the model.

## 1 INTRODUCTION

Forecasting in macroeconomics and finance requires flexible models that are capable of capturing salient features of the data such as structural breaks in the regression coefficients and/or heteroscedastic measurement errors. Time variation in the shocks is often introduced through stochastic volatility (SV) models that imply a smoothly evolving error variance over time. Such models typically rule out that the level of the volatility directly affects the conditional mean of the predictive regression. This assumption is relaxed in Koopman and Hol Uspensky (2002) and Chan (2017) by assuming that the volatilities enter the conditional mean equation and thus exert a direct effect on the quantity of interest.

In this article, we reconsider the model proposed in Chan (2017) and replicate the main findings both in a narrow and in a wide sense. The original specification is a time‐varying parameter (TVP) model with SV that allows for feedback effects between the level of volatility and the endogenous variable. As opposed to most of the existing literature, this model assumes that this relationship is time varying. Estimation and inference are performed in a Bayesian framework, and this implies that prior distributions are specified on all coefficients of the model. These priors are often set to be weakly informative.

One key contribution of this paper is to introduce shrinkage via state‐of‐the‐art dynamic shrinkage priors that allow for capturing situations where coefficients are time varying over certain periods in time while they remain constant in others. These priors are based on a recent paper (Kowal, Matteson, & Ruppert, 2019) that proposes a dynamic shrinkage process that is time varying and follows an AR(1) model with *Z*‐distributed shocks. Proper specification of the hyperparameters of this error distribution yields a dynamic horseshoe (DHS) prior that possesses excellent shrinkage properties. Other specifications we propose also introduce shrinkage but assume the shrinkage coefficients to be independent over time (static horseshoe [SHS] prior) or 1 time invariant, such as a standard horseshoe (HS) prior that exploits the noncentered parameterization of the state‐space model (see Frühwirth‐Schnatter & Wagner, 2010).

The second contribution deals with replicating the main findings of Chan (2017) using updated real‐time inflation data. Instead of considering the original three countries (the United States, the United Kingdom, and Germany), we replace Germany with the euro area (EA) and investigate whether the main findings also hold for this dataset. Using more flexible shrinkage priors generally yields similar in‐sample findings for the United States and the United Kingdom. For the EA, we find only minor evidence of a link between inflation and inflation volatility. This finding relates to Jarociński and Lenza (2018), who observe limited evidence in favor of SV for inflation derived from the harmonized index of consumer prices (HICP). When it comes to forecasting, we find that shrinkage sometimes improves predictive accuracy. In cases where predictive accuracy is below the no‐shrinkage specification, these differences are often very small.

In the remainder of the article, we proceed as follows. The next section summarizes the model and motivates our shrinkage priors. Section 3 replicates the main findings of Chan (2017) using the proposed model and carries out a real‐time forecasting exercise to show that using shrinkage often further improves upon the already excellent predictive performance of the original model. The last section briefly summarizes and concludes the paper.

## 2 ECONOMETRIC FRAMEWORK

### 2.1 The TVP‐SVM model

The time‐varying parameter stochastic volatility in mean (TVP‐SVM) model is given by

(1) $$y_{t}=\tau_{t}+\beta_{t}^{\prime }z_{t}+\gamma_{t}e^{h_{t}}+\epsilon_{t},\;\epsilon_{t}∼N\left(0,e^{h_{t}}\right),$$

(2) $$h_{t}=\mu_{h}+\phi_{h}(h_{t-1}-\mu_{h})+\delta y_{t-1}+\nu_{t},\;\nu_{t}∼N(0,\sigma^{2}),$$

where *y*_*t*_ is a scalar time series, *τ*_*t*_ denotes a stochastic trend term, ***β***_*t*_ is a *K*‐dimensional vector of dynamic regression coefficients, and *γ*_*t*_ is a coefficient that measures the (potentially) time‐varying relationship between *y*_*t*_ and the shock volatility $e^{h_{t}}$. The column vector ***z***_*t*_ may contain lags of the dependent variable, additional predictors, and/or latent factors capturing high‐dimensional information. The log volatility *h*_*t*_ follows an AR(1) process with unconditional mean *μ*_*h*_, persistence parameter *ϕ*_*h*_, and error variance *σ*^2^. *h*_*t*_, moreover, depends on the lag of *y*_*t*_ through a time‐invariant parameter *δ*.

Let $x_{t}={\left(1,z_{t}^{\prime },e^{h_{t}}\right)}^{\prime }$ and $\theta_{t}={\left(\tau_{t},\beta_{t}^{\prime },\gamma_{t}\right)}^{\prime }$ of size *k* × 1 (with k=2+K), then Equation (1) can be written in regression form:

(3) $$y_{t}=\theta_{t}^{\prime }x_{t}+\epsilon_{t},\;\epsilon_{t}∼N\left(0,e^{h_{t}}\right).$$

Furthermore, we assume that ***θ***_*t*_ evolves according to a random walk (RW):

(4) $$\theta_{t}=\theta_{t-1}+e_{t},\;e_{t}∼N(0,\Omega ),$$

with Gaussian errors and diagonal covariance matrix $\Omega =\text{diag}(\omega_{1},\ldots ,\omega_{k})$.

### 2.2 Imposing shrinkage in TVP models

The model outlined in the previous subsection is quite flexible and allows for a direct relationship between the error volatilities and *y*_*t*_. This relationship might be subject to parameter instability. Allowing for TVPs in all coefficients could, however, lead to overfitting, and this often decreases predictive accuracy. Chan (2017) uses weakly informative priors on key parameters and finds them to yield good forecasting results.

Here, we aim to improve upon this finding by introducing three additional priors that allow us to flexibly select restrictions in the empirical model and thus achieve parsimony. The priors we consider in this study are given by

1. A weakly informative prior on the coefficients and state innovation variances similar as in Chan (2017). We use independent weakly informative inverse Gamma priors on the innovation variances of the state equation $\omega_{j}\;(j=1,\ldots ,k)$. We subsequently label this prior “None,” reflecting the notion that almost no shrinkage is imposed.
2. A hierarchical global local prior on the constant part and innovation variances of the model. We achieve this by rewriting the model in the noncentered parameterization of Frühwirth‐Schnatter and Wagner (2010):
  (5) $$y_{t}=\theta_{0}^{\prime }x_{t}+{\tilde{\theta }}_{t}^{\prime }\sqrt{\Omega }x_{t}+\epsilon_{t},$$
  (6) $${\tilde{\theta }}_{t}={\tilde{\theta }}_{t-1}+\eta_{t},\;\eta_{t}∼N(0_{k},I_{k}),$$
  with $\sqrt{\Omega }=\text{diag}\left(\sqrt{\omega_{1}},\ldots ,\sqrt{\omega_{k}}\right)$, the *j*th element of ${\tilde{\theta }}_{jt}=(\theta_{jt}-\theta_{j0})/\sqrt{\omega_{j}}$ and ${\tilde{\theta }}_{0}=0_{k}$. We collect the constant parameters and the state innovation variances in a 2*k* × 1‐vector $\alpha ={\left(\theta_{0}^{\prime },\sqrt{\omega_{1}},\ldots ,\sqrt{\omega_{k}}\right)}^{\prime }$ and index its *i*th element for i=1,…,2k by *α*_*i*_. Any shrinkage prior on these coefficients may be used. We rely on the popular horseshoe prior (labeled “HS” in the empirical application) of Carvalho, Polson, and Scott (2010) in its auxiliary representation (Makalic & Schmidt, 2015):
  (7) $$\alpha_{i}∼N(0,\phi_{i}\lambda ),\;\phi_{i}∼G^{-1}(1/2,1/v_{i}),\;\lambda ∼G^{-1}(1/2,1/w),$$
  with $v_{i}∼G^{-1}(1/2,1)$ for i=1,…,2k and $w∼G^{-1}(1/2,1)$. Here, $G^{-1}$ denotes the inverse Gamma distribution. 2
3. A static variant of the horseshoe prior (labeled “SHS”) that imposes shrinkage using the centered parameterization of the state equation with time‐varying variances:
  (8) $$\theta_{t}=\theta_{t-1}+e_{t},\;e_{t}∼N(0,\Omega_{t}).$$
  We denote the *j*th diagonal element of **Ω**_*t*_ by $\omega_{jt}=\lambda_{j}\phi_{jt}$ and assume inverse Gamma distributions as priors for the global and local shrinkage parameters
  (9) $$\phi_{jt}∼G^{-1}(1/2,1/v_{jt}),\;\lambda_{j}∼G^{-1}(1/2,1/w_{j}).$$
  Following Makalic and Schmidt (2015), auxiliary variables $v_{jt}∼G^{-1}(1/2,1)$ and $w_{j}∼G^{-1}(1/2,1)$ for j=1,…,k are used for establishing the horseshoe prior. Here, *λ*_*j*_ governs the overall amount of time variation for the coefficient of the *j*th regressor, whereas *ϕ*_*jt*_ allows for predictor and time‐specific shrinkage.
4. A dynamic horseshoe prior (labeled “DHS”) as in Kowal et al. (2019). Again using the centered parameterization of the state equation with time‐varying state innovation variances in **Ω**_*t*_ with *j*th element $\omega_{jt}=\lambda_{0}\lambda_{j}\phi_{jt}$. To achieve a log‐scale representation of the global local prior, define $\psi_{jt}=\log(\lambda_{0}\lambda_{j}\phi_{jt})$ and assume
  (10) $$\psi_{jt}=\mu_{\psi j}+\varphi_{j}(\psi_{t-1}-\mu_{\psi j})+\nu_{jt},\;\nu_{jt}∼Z(a,b,0,1),$$
  with Z denoting the *Z*‐distribution, where setting a=b=1/2 yields the DHS prior (for details on related prior choices, see Kowal et al. 2019). Here, *λ*_0_ is a global, *λ*_*j*_ are predictor specific, and *ϕ*_*jt*_ are predictor and time‐specific shrinkage parameters that follow a joint autoregressive law of motion.

We use standard Markov chain Monte Carlo (MCMC) methods such as Gibbs sampling augmented by a forward filtering backward sampling (FFBS) algorithm for the TVPs (Carter & Kohn, 1994; Frühwirth‐Schnatter, 1994). For the log volatilities related to the dynamic shrinkage prior, the procedure outlined in Kowal et al. (2019) employing a mixture representation of the *Z*‐distribution using Pólya–Gamma random variables is applicable.

The SVM specification makes it impossible to linearize the respective SV state equation required for the conventional auxiliary mixture approximation sampler (see Kim, Shephard, & Chib, 1998). Here, we rely on independent Metropolis–Hastings updates discussed as an alternative by Kim et al. (1998, p. 365), adapted for the SVM case. We use a prior setup for the SV state equation similar to Kastner and Frühwirth‐Schnatter (2014). Our algorithm is implemented in R, and further details are provided in Appendix A. The implementation in R serves to provide further robustness to the findings from the MATLAB implementation in the original contribution.

## 3 INFLATION MODELING

In this study, we take a real‐time perspective to modeling inflation for the United States, the United Kingdom, and the EA. Vintage data available at specific times in the past are obtained from the webpages of the Federal Reserve Bank of Philadelphia (philadelphiafed.org), the Bank of England (bankofengland.co.uk), and the European Central Bank (sdw.ecb.europa.eu).

Price indices *p*_*t*_ taken from the respective databases are seasonally adjusted and on quarterly frequency (taking the average over the respective months if on higher frequency originally). We use the consumer price index (CPIAUCSL) for the United States, the gross domestic product deflator at market prices (PGDPDEF) for the United Kingdom, and the HICP for the EA. Historical vintage data for the United States, the United Kingdom, and the EA start in 1994, 1990, and 2001, resulting in differently sized natural holdout samples with a total available time period ranging from 1947:Q1 to 2019:Q4 (the United States), 1970:Q1 to 2016:Q3 (the United Kingdom), and 1990:Q1 to 2019:Q1 (the EA), respectively.

We model inflation, defined as $\pi_{t}=400\log(p_{t}/p_{t-1})$, with an unobserved component model augmented with stochastic volatility in the mean (UC‐SVM):

$$\begin{array}{ccc} \pi_{t} & =\tau_{t}+\gamma_{t}e^{h_{t}}+\epsilon_{t},\;\epsilon_{t}∼N(0,e^{h_{t}}), & \\ h_{t} & =\mu_{h}+\phi_{h}(h_{t-1}-\mu_{h})+\delta \pi_{t-1}+\nu_{t},\;\nu_{t}∼N(0,\sigma^{2}), \end{array}$$

which is a special case of Equation (1) with $\beta_{t}=0$ for all *t*. This model has been used by Chan (2017) to forecast inflation. If $\gamma_{t}=0$, we obtain the unobserved component model augmented with stochastic volatility (UC‐SV) model proposed in Stock and Watson (2007). If the prior on the state innovation variances is specified too loose, the model might be prone to overfitting, and this would be deleterious for predictive accuracy. Hence, in this empirical application, we assess whether using shrinkage priors improves the predictive fit of the model, but before we turn to analyzing predictions, we focus on key in‐sample results.

### 3.1 In‐sample results

**FIGURE 1:**
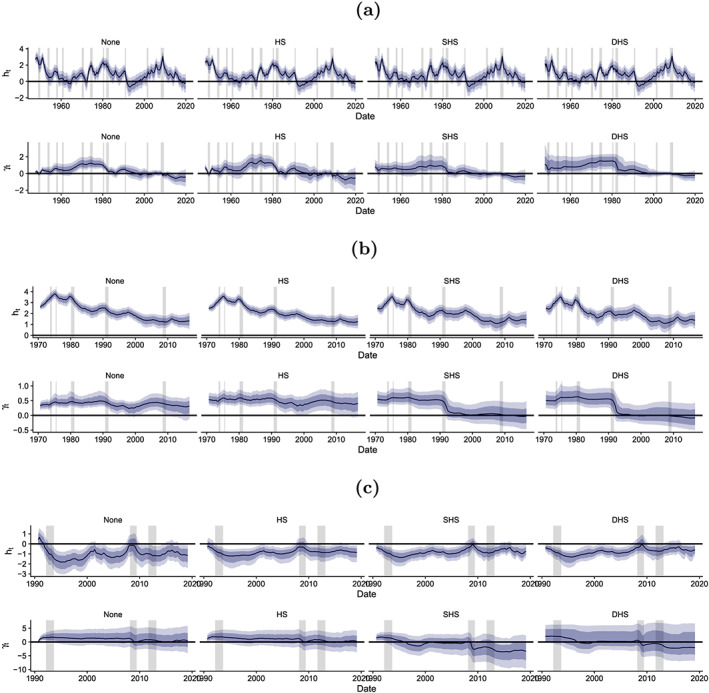
Time‐varying volatilities *h*_*t*_ and associated regression coefficients *γ*_*t*_. The black line is the posterior median estimate, alongside the 68% (dark blue) and 90% (light blue) posterior credible sets. Recessions are indicated as gray vertical bars. (a) United States, (b) United Kingdom, and (c) euro area [Colour figure can be viewed at wileyonlinelibrary.com]

Figure 1 shows selected posterior credible intervals for the time‐varying volatilities *h*_*t*_ and the corresponding time‐varying regression coefficients *γ*_*t*_ over the full estimation period and across the three considered economies.

For the United States and the United Kingdom, the main impression is that the specific choice of the shrinkage specification plays only a minor role for the estimates of *h*_*t*_. In the case of the EA, the prior seems to have some impact on the log volatilities. In this case, any of the shrinkage priors appreciably reduces time variation in *h*_*t*_ for most periods except for the global financial crisis (GFC) in 2008/2009. Before and after that period, the error volatility process remains rather stable (as opposed to more rapidly changing log volatilities in the no‐shrinkage case).

Turning to the findings for *γ*_*t*_ yields a different picture. Although low‐frequency movements remain similar across shrinkage priors, some interesting differences arise. Shrinkage specifications that imply time‐varying shrinkage (i.e., SHS and DHS) allow for sharp movements in *γ*_*t*_ for selected periods and across economies. For instance, in the United States, we observe a pronounced change in the relationship between inflation and inflation volatility during the Volcker disinflation in 1981/1982. A comparable appreciable decrease in *γ*_*t*_ can also be observed in the United Kingdom during the crisis of the European Exchange Rate Mechanism (ERM) in 1992, after which the Bank of England adopted inflation targeting. A similar decline, albeit more noisy, is evident during the GFC in the EA, a period where several new unconventional monetary policy instruments were introduced.

In sum (and with some exceptions), Figure 1 shows that the original results of Chan (2017) remain remarkably robust with respect to different shrinkage priors. Exceptions arise especially during periods where the level of inflation experienced sharp changes (such as during the Volcker disinflation, the crisis of the ERM and the GFC) and for EA data.

Cukierman and Meltzer (1986) provide an explanation for our findings from a theoretical perspective. Using a stylized model, they show that high inflation uncertainty can subsequently stimulate inflation. This relationship is subject to change during policy‐related regime changes at central banks. Our results corroborate the findings of Chan (2017) in this context. The SVM model combined with novel shrinkage priors clearly supports the theoretical predictions and does so with more precise statistical inference.

To conclude this section on in‐sample results, we now compare how the different shrinkage priors affect the in‐sample fit of the model. Here, we rely on particle filtering using 10,000 particles (see, for instance, Fernández‐Villaverde, 2010) to calculate the observed‐data likelihood of our model. We use this quantity to construct the deviance information criterion (DIC, Spiegelhalter et al. 2002), a measure rewarding model fit while penalizing model complexity. 3

The results are provided in Table 1. Assessing the DIC for US data shows that the weakly informative prior yields the best fit, followed by the HS prior in the noncentered TVP parameterization. For the United Kingdom, the DIC favors shrinkage in form of the DHS prior (with insignificant estimates of *γ*_*t*_ after the ERM crisis). The case of the EA yields a different picture. Here, the DIC selects the HS prior as the clearly superior specification. The SHS prior resulting in a negative relationship between inflation and inflation volatility after the GFC exhibits the least favorable metric. Whereas the DIC varies substantially across priors for the United States and the EA, these differences are muted for UK data.

**TABLE 1:** Model selection via the deviance information criterion.

Prior/Economy	United States	United Kingdom	Euro area
None	1942.200	1200.909	1020.483
HS	2049.749	1207.098	704.642
SHS	4911.401	1224.727	3593.571
DHS	3025.361	1198.502	1187.478

*Note*: Deviance information criterion (DIC, Spiegelhalter, Best, Carlin, & Van Der Linde, 2002) for the unobserved component stochastic volatility in mean models estimated with different shrinkage priors. Smaller values are superior for the DIC.

Abbreviations: DHS, dynamic horseshoe; HS, horseshoe; SHS, static horseshoe.

### 3.2 Forecast results

In this section, we analyze whether our set of shrinkage priors improves out‐of‐sample predictive performance within a real‐time forecasting exercise. We evaluate both point and density forecasts by means of root mean squared errors (RMSEs) and average log‐predictive likelihoods (LPLs).

Each real‐time vintage (i.e., the training samples) is used to produce forecasts, which are then evaluated using the final available vintage (actual realizations of the series in the holdout period). We denote the data in the respective vintage up to time *t* by ***y***_1 : *t*_ and use these data to estimate the posterior distributions of the parameters of our model. This enables using simulation methods to calculate the *h*‐step ahead predictive density *p*(*y*_*t* + *h*_|***y***_1 : *t*_) and the predictive mean $𝔼(y_{t+h}|y_{1:t})$. In line with Chan (2017), we analyze one‐quarter ( h=1) and one‐year ahead ( h=4) forecasts.

Let *T*_*H*_ denote the length of the holdout sample and $y_{t+h}^{\left(r\right)}$ the realization of the series in the holdout. RMSEs for *h*‐step ahead forecasts are defined as

$${\text{RMSE}}_{h}=\sqrt{\overset{T_{H}}{\underset{t=T}{\sum }}{\left(y_{t+h}^{\left(r\right)}-𝔼(y_{t+h}|y_{1:t})\right)}^{2}/(T_{H}-T)}.$$

This measure captures average deviations from the realizations over the holdout. For evaluating predictive densities, we calculate LPLs:

$${\text{LPL}}_{t+h}=\log\left(p\left(y_{t+h}^{\left(r\right)};y_{t+h}|y_{1:t}\right)\right).$$

The expression $p(y_{t+h}^{\left(r\right)};y_{t+h}|y_{1:t})$ denotes evaluating the realized value in the predictive density. As opposed to point forecast evaluation, LPLs take into account higher order moments of the predictive distribution. Average LPLs are computed by taking the arithmetic mean of the LPLs over *t* in the holdout for each horizon *h*. Note that the sum of LPLs is closely related to the marginal likelihood, conditional on the initial estimation sample (see also Geweke & Amisano, 2010).

We assess the merits of using shrinkage in the SVM model relative to the following competitors. As in Chan (2017), we use a RW model as the benchmark for relative RMSEs and LPLs: $\pi_{t}=\pi_{t-1}+\eta_{t}$, with $\eta_{t}∼N(0,\sigma_{\eta }^{2})$. Moreover, we include UC‐SV as a special case of the UC‐SVM model: $\pi_{t}=\tau_{t}+\epsilon_{t}$. We assume $\epsilon_{t}∼N(0,e^{h_{t}})$ with the state equation given by $h_{t}=\mu_{h}+\phi_{h}(h_{t-1}-\mu_{h})+\nu_{t}$ and $\nu_{t}∼N(0,\sigma^{2})$. UC‐SV and UC‐SVM are estimated using the four shrinkage priors (None, HS, SHS, and DHS) discussed above. Relative RMSEs are calculated as ratios to the benchmark (lower ratios indicate superior performance), whereas relative average LPLs are presented in differences (larger numbers are superior).

Table 2 presents forecasting results for different economies and shrinkage priors. In general (and with only very few exceptions), we find that all models improve upon the RW. This holds true for both point and density forecasts, all economies and forecast horizons considered. Only in the case of density forecast accuracy (in terms of LPLs) for EA inflation we find the RW to yield more precise predictions except for the HS prior. The strong performance of the UC‐SVM model *without* shrinkage confirms the findings reported in Chan (2017).

**TABLE 2:** Predictive inference relative to the benchmark model

	RMSE		LPL	
Model	UC‐SV	UC‐SVM	UC‐SV	UC‐SVM
United States	*One‐quarter ahead*			
None	0.677	0.726	0.374	0.499
HS	0.679	0.777	0.474	0.477
SHS	0.678	0.737	0.370	0.489
DHS	0.680	0.724	0.369	0.488
	*One‐year ahead*			
None	0.742	0.757	0.467	0.584
HS	0.740	0.788	0.539	0.570
SHS	0.743	0.768	0.453	0.592
DHS	0.739	0.764	0.448	0.572
United Kingdom	*One‐quarter ahead*			
None	0.924	0.803	0.100	0.313
HS	0.807	0.810	0.245	0.289
SHS	0.818	0.805	0.122	0.305
DHS	0.832	0.806	0.126	0.305
	*One‐year ahead*			
None	0.915	0.820	0.612	0.873
HS	0.796	0.868	0.764	0.856
SHS	0.803	0.865	0.636	0.849
DHS	0.823	0.873	0.638	0.850
Euro area	*One‐quarter ahead*			
None	0.859	0.789	−0.294	0.000
HS	0.837	0.816	0.125	0.108
SHS	0.865	0.821	−0.291	0.061
DHS	0.867	0.815	−0.297	0.091
	*One‐year ahead*			
None	0.765	0.868	−0.140	0.094
HS	0.774	0.858	0.228	0.204
SHS	0.764	0.885	−0.140	0.175
DHS	0.771	0.864	−0.146	0.205

*Note*: All measures are relative to the random walk benchmark. RMSEs are ratios (smaller numbers indicate superior performance), and average LPLs are differences (larger numbers indicate superior performance).

Abbreviations: DHS, dynamic horseshoe; HS, horseshoe; LPL, log‐predictive likelihood; RMSE, root mean squared error; SHS, static horseshoe; UC‐SV, unobserved component model with stochastic volatility; UC‐SVM, unobserved component stochastic volatility in mean model.

We now investigate whether using shrinkage further improves predictive accuracy. Considering both density and point forecasts, this question is difficult to answer. For some economies, horizons, and specifications, shrinkage priors seem to improve both point and density forecasting performance, whereas for other configurations, shrinkage seems to slightly hurt predictive accuracy, but these differences (both negative and positive) are often very small. There exist some cases where we find more pronounced improvements. For instance, the UC‐SV model with shrinkage performs appreciably better in predicting UK inflation at both horizons and by considering RMSEs and LPLs than the no‐shrinkage counterpart. Another example that provides evidence that shrinkage improves forecasts can be found for EA inflation density forecasts. In this case, any shrinkage prior yields better forecasts than the no‐shrinkage specification.

Considering differences between the different shrinkage priors provides no clear winner of our forecasting horse race. In most cases, predictions are similar to each other. If we were to choose a preferred prior, our default recommendation would be the HS specification. This is because it performs well across the different configurations and for both model classes considered. Especially in the case of the EA, we find the HS setup to provide favorable point and density forecasts (especially for the UC‐SV model). It is worth mentioning that these out‐of‐sample forecast results roughly correspond to our findings in terms of model selection based on the DIC.

The key takeaway from this discussion is that the benchmark model introduced in Chan (2017) seems to work very well for all considered economies. Using shrinkage not only helps in some cases but also leads to slightly inferior predictive performance in others. However, these decreases in forecast accuracy are never substantial. By contrast, we observe several cases where shrinkage improves forecasts, and these improvements are substantial. Hence, as a general rule, we can suggest combining the SVM model with shrinkage priors because the risk of obtaining markedly weaker forecasts appears to be low, whereas the chances that forecasts can be improved substantially are much higher.

## 4 CONCLUDING REMARKS

In this paper, we have successfully replicated the findings in Chan (2017) both in a narrow and in a wide sense. We have shown that using several different shrinkage techniques has the potential to improve forecasts. Although these gains are small on average, several cases emerge where improvements are more pronounced. More importantly, we never find situations where using shrinkage strongly decreases forecast performance.

### OPEN RESEARCH BADGES

This article has been awarded Open Data Badge for making publicly available the digitally‐shareable data necessary to reproduce the reported results. Data is available at [http://qed.econ.queensu.ca/jae/datasets/huber005/]

## Supporting information

The JAE Data Archive directory is available at http://qed.econ.queensu.ca/jae/datasets/huber005/

## Footnotes

1 A similar exercise using a mixture innovation model is provided in Hou (2020).

2 The inverse Gamma distribution $G^{-1}(a,b)$ is parameterized such that its probability density function is $p(z|a,b)=b^{a}/\Gamma (a)z^{-a-1}\exp(-b/z)$.

3 We calculate the DIC using the observed‐data likelihood rather than the complete‐data likelihood of the state‐space model. This is due to the known issue of the DIC based on the complete‐data likelihood often selecting overfitting models (see Chan & Grant, 2016).

## APPENDIX A SAMPLING ALGORITHM

### A.1

After initializing the parameters of our model, we use a comparatively standard Markov chain Monte Carlo (MCMC) algorithm involving Gibbs sampling, forward filtering backward sampling (FFBS) for the time‐varying parameters (TVPs), and an independent Metropolis–Hastings (MH) step for the stochastic volatilities (SVs). To preserve space, we refrain from reproducing all posterior distributions but refer to several papers from where they can be obtained. Our algorithm iterates through the following steps:

1. Conditional on the data, the full history of the state innovation variances, and the log volatilities, as well as all other parameters of the model, we produce a draw for the full history of the TVPs using an FFBS algorithm (see Carter & Kohn, 1994; Frühwirth‐Schnatter, 1994).
2. Conditional on a draw of the full history of the latent states, the step to draw the state innovation variances depends on the respective prior:
  - *None*: The inverse Gamma prior translates to the well‐known posterior distribution for error variances in a linear Bayesian regression model (see, for instance, Koop, 2003).
  - *HS*: Using the noncentered parameterization of the model allows for drawing the constant part of the parameters and the state innovation variances conditionally in one step using standard posteriors for a linear Bayesian regression model. For details, see Frühwirth‐Schnatter and Wagner (2010). The shrinkage parameters are drawn from their respective inverse Gamma posteriors; see Makalic and Schmidt (2015).
  - *SHS*: We draw the global and local shrinkage parameters alongside the auxiliary variables from their inverse Gamma posterior distributions; see Makalic and Schmidt (2015).
  - *DHS*: We draw the full history of the state innovation variances employing the algorithm proposed and discussed in Kowal et al. (2019).
3. For producing a draw of the full history of the SVs that also feature in the mean of the process, we rely on an adapted version of the independent MH procedure discussed by Kim et al. (1998, p. 365). Here, the idea is to construct a proposal density based on a Taylor series expansion of the conditional likelihood. Using the proposal, we can compute its acceptance probability, taking into account that the volatility also affects the mean of the process.
4. Conditional on the full history of the SVs, the parameters in the state equation of the SVs are drawn based on the posteriors provided in Kastner and Frühwirth‐Schnatter (2014).
5. If the observed series features ragged edges at the end of the real‐time vintages, we use a data augmentation step and draw the missings conditional on all other parameters of the model.
